# Optical gradient force on chiral particles

**DOI:** 10.1126/sciadv.abq2604

**Published:** 2022-09-21

**Authors:** Junsuke Yamanishi, Hyo-Yong Ahn, Hidemasa Yamane, Shun Hashiyada, Hajime Ishihara, Ki Tae Nam, Hiromi Okamoto

**Affiliations:** ^1^ Institute for Molecular Science, National Institutes of Natural Sciences, 38 Nishigonaka, Myodaiji, Okazaki, Aichi 444-8585, Japan.; ^2^ Center for Novel Science Initiatives, National Institutes of Natural Sciences, 4-3-13 Toranomon, Minato-ku, Tokyo 105-0001, Japan.; ^3^ Department of Physics and Electronics, Osaka Prefecture University, 1-1 Gakuen-cho, Naka-ku, Sakai, Osaka 599-8531, Japan.; ^4^ Department of Physics, Kitasato University, 1-15-1 Kitasato, Minami-ku, Sagamihara, Kanagawa 252-0373, Japan.; ^5^ Innovative Photon Manipulation Research Team, RIKEN Center for Advanced Photonics, 2-1 Hirosawa, Wako, Saitama 351-0198, Japan.; ^6^ Department of Electrical, Electronic, and Communication Engineering, Faculty of Science and Engineering, Chuo University, 1-13-27 Kasuga, Bunkyo-ku, Tokyo 112-8551, Japan.; ^7^ Department of Materials Engineering Science, Osaka University, 1-3 Machikaneyama-cho, Toyonaka, Osaka 560-8531, Japan.; ^8^ Center for Quantum Information and Quantum Biology, Osaka University, 2-1 Yamadaoka, Suita, Osaka 565-0871, Japan.; ^9^ Department of Materials Science and Engineering, Seoul National University, 1 Gwanak-ro, Gwanak-gu, Seoul 08826, Republic of Korea.

## Abstract

When a chiral nanoparticle is optically trapped using a circularly polarized laser beam, a circular polarization (CP)–dependent gradient force can be induced on the particle. We investigated the CP-dependent gradient force exerted on three-dimensional chiral nanoparticles. The experimental results showed that the gradient force depended on the handedness of the CP of the trapping light and the particle chirality. The analysis revealed that the spectral features of the CP handedness–dependent gradient force are influenced not only by the real part of the refractive index but also by the electromagnetic field perturbed by the chiral particle resonant with the incident light. This is in sharp contrast to the well-known behavior of the gradient force, which is governed by the real part of the refractive index. The extended aspect of the chiral optical force obtained here can provide novel methodologies on chirality sensing, manipulation, separation, enantioselective biological reactions, and other fields.

## Teaser

Optical trapping force of chiral nanoparticle was dependent on the handedness of incident circularly polarized light.

## INTRODUCTION

Chirality is a geometrical property that dictates that matter cannot be superposed on its mirror image. Chiral materials exhibit chiro-optical effects, which have different interactions with left and right circularly polarized (LCP and RCP) light (*1*–*7*). The chiro-optical effects of chiral molecules and nanoparticles can be observed with optical polarization measurements, such as circular dichroism (CD) and optical rotation (OR) (*8*, *9*). Chiro-optical effects can also influence the motion of chiral materials illuminated by circularly polarized (CPd) light when an optical force acts on the particles (*4*, *10*–*13*). In previous studies, the optical scattering forces induced by LCP and RCP light were reported to act differently on the micrometer-scale droplets of liquid crystals composed of chiral molecules (*14*, *15*). Although these reports presented a novel approach for investigating chiral optical forces, their relation with chiro-optical effects has yet to be fully revealed. We can also expect that the optical force exerted by CPd light on a chiral particle with a diameter of less than half the wavelength of light will influence the motion of the particle (*11*). A lot of theoretical studies have reported the optical force relating to the chirality of nanoparticles (*10*–*13*, *16*–*20*). By establishing a more detailed relationship between the chiro-optical properties of nanoparticles and CP-dependent optical forces, including scattering and gradient forces, we can greatly extend the potential for optical manipulations of nanomaterials.

In the present study, we investigated the chiral characteristics of gradient forces for chiral nanoparticles, which had not previously been studied experimentally. We analyzed the mechanical behavior of chiral gold nanoparticles (*21*), which exhibit strong chiro-optical effects when optically trapped by CPd light. Chiral gold nanoparticles can be used to represent chiral nanomaterials and molecules with dimensions smaller than the wavelength of the interacting light. We observed the chiral characteristics of the gradient force on the nanoparticles and discussed the underlying mechanisms. We observed that the strength of the gradient force differed under LCP illumination and RCP illumination in the wavelength region where the particles exhibited strong chiro-optical effects. The strength of the gradient force was estimated on the basis of the position fluctuation (dispersion) σ of the trapped particle due to Brownian motion. The difference between the position dispersion under LCP trapping and that under RCP trapping (σ_L_ − σ_R_) was found to be as much as approximately 20 % of the average dispersion ((σ_L_ + σ_R_)/2), depending on the wavelength of the light. The correlation between the spectral features of the chiral trapping force and those of the chiro-optical effect (CD and OR) of the particle was also elucidated.

To discuss the optical gradient force acting on a chiral particle, we assumed that the particle was isotropic and sufficiently smaller than half the wavelength of the incident light. The induced polarization is expressed as follows (*10*, *13*, *17*)

p=αE+iχH (1)

where α and χ denote the electric and cross-term (electric-magnetic) polarizabilities of the isotropic particle, respectively. Note that χ is related to the chirality of the particles (*22*). Although these quantities are of tensor character in origin, we assume that, here, the anisotropy of the particle is small and the polarizabilities are treated as scalar quantities for simplicity. ***E*** and ***H*** represent the total electric and magnetic fields, respectively. Then, the time-averaged gradient force exerted on the particle can be expressed as (*13*, *23*)

$$<F_{\text{grad}}>=\frac{1}{4}\nabla (\text{Re}[\alpha ]{|E|}^{2}-2\text{Re}[\chi ]\text{Im}[H\cdot E*])$$ (2)

Conventional nanoparticle optical trapping arises from the first term in the bracket in Eq. 2 (*24*, *25*). The second term must be considered if the particle is chiral; this term is proportional to the optical chirality of the field, which is defined as 𝒞 = − (1/2)ωϵ_0_ Im [***B*** · E^*^] (*4*), where ω and ϵ_0_ are the angular frequency of the light and the vacuum permittivity, respectively, and ***B*** denotes the magnetic flux density. In general, Re[χ] is much smaller than Re[α] for small materials (*4*), and the gradient force originating from the second chirality is hardly observable. However, chiral materials that exhibit a strong chiro-optical effects, such as the chiral gold nanoparticles investigated here (*21*, *26*), have large Re[χ] values in the wavelength region with the chiral optical transitions. The second term in Eq. 2 is thus expected to contribute substantially to the gradient force.

**Fig. 1.**
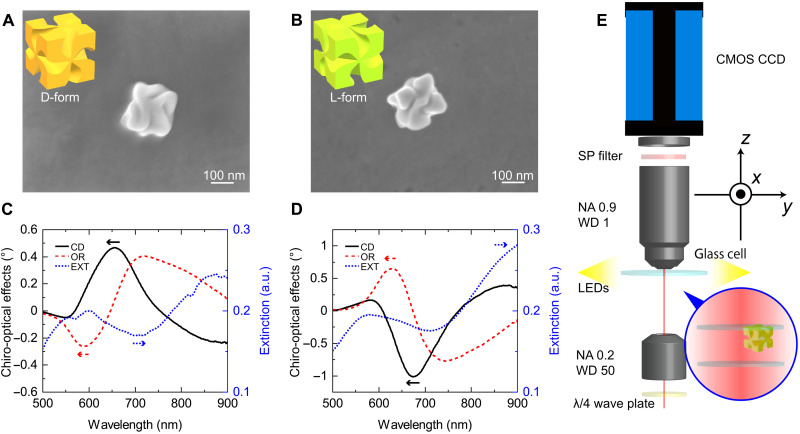
Measurements of the chiral gold nanoparticles. (**A** and **B**) SEM images of D-form and L-form chiral gold nanoparticles, respectively. (**C** and **D**) Chiro-optical effects (CD and OR) and extinction (EXT) spectra of left- and right-handed chiral nanoparticles in colloidal solutions, respectively. a.u., arbitrary units. (**E**) Schematic illustration of the experimental optical trapping system. NA, numerical aperture; WD, working distance (mm); SP, short pass.

The chiral gold nanoparticles were fabricated with solution chemical synthesis (*21*). The morphologies of the synthesized D- and L-form particles were imaged by scanning electron microscopy (SEM), as shown in Fig. 1 (A and B), respectively. On the basis of these SEM images, the base lengths of the particles were estimated to be ∼190 nm. The chiro-optical effects (CD and OR) and the extinction spectra of the particles are shown in Fig. 1 (C and D), respectively. As shown in Fig. 1 (C and D), the chiro-optical effect appeared for the plasmonic modes in the wavelength region between 550 and 750 nm. The optical gradient force was expected to depend on the handedness of the incident CPd light in this wavelength region. We optically trapped the chiral nanoparticles with the experimental setup depicted in Fig. 1E. The trapping laser was incident on the bottom of the system. The polarization of the trapping laser light can be adjusted by the λ/4 wave plate mounted just below the bottom objective lens. In free space, the strong scattering force prevents the optical trapping of the nanoparticle in the focal spot, when the incident wavelength is resonant with the plasmon mode. This is because the scattering force pushes the nanoparticle in the direction of the incident light propagation, releasing the particle from the trapping. To avoid this, we prevented the gold nanoparticle from moving in the *z* direction with a cover glass, trapping the particle in the focal spot area in the *xy* plane. The trapped particle was illuminated by light-emitting diodes (LEDs) from the side of the sample glass cell and observed by a complementary metal-oxide semiconductor (CMOS) camera after passing through the top objective lens and the short-pass filter (see the Supplementary Material for additional experiment details).

## RESULTS

### Measurement of Brownian motion

**Fig. 2.**
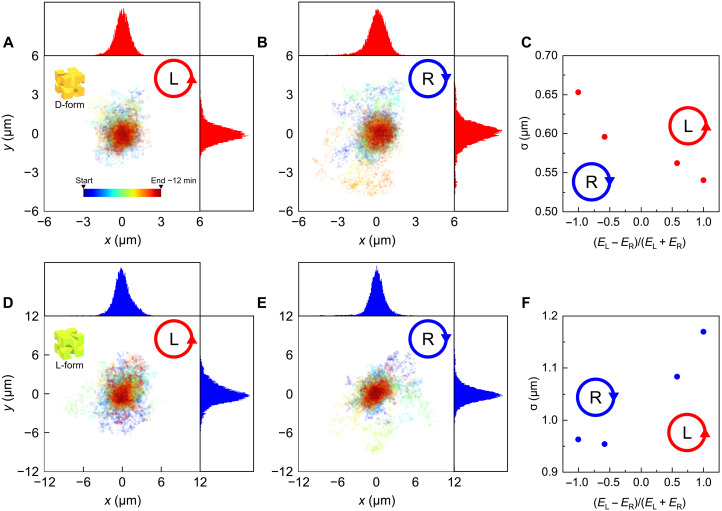
Measurement of the Brownian motions of the nanoparticles. (**A** and **B**) Position traces for the D-form particle under LCP and RCP illumination, respectively. The colors of the dots indicate the passage of time. (**C**) Position dispersion σ for the D-form particle as a function of the ellipticity of the light, (*E*_L_ − *E*_R_)/(*E*_L_ + *E*_R_), where *E*_L_ and *E*_R_ are the absolute amplitudes of the LCP and RCP components of the electric field, respectively. (**D** and **E**) Position traces for the L-form particle. (**F**) Position dispersion for the L-form particle as a function of the ellipticity of the light. The intensities of the incident laser were 15 and 34 mW for the D-form (A to C) and L-form (D to F) particles, respectively.

In Fig. 2 (A, B, D, and E), the recorded lateral positions in the *xy* plane of the trapped particle in each frame of the video images are plotted, under the illumination of CPd light at a wavelength of 680 nm, with the CD signals showing nearly extremal values [see Fig. 1 (C and D)]. The dot colors in the plots indicate the passage of time. As shown in these plots, the positions of the particles were extended to a certain area because of Brownian motion. The histograms in each axis direction (*x* and *y*) were fit to a Gaussian function ($A\text{exp}\;[-{|r-r_{0}|}^{2}/(2\sigma_{r}^{2})];r=x,y;r_{0}=x_{0},y_{0}$), and the fitting parameters of *A*, *r*_0_, and σ*_r_* were estimated (*27*). For the D-form particle (Fig. 2, A and B), the average dispersion (σ = (σ*_x_* + σ*_y_*)/2) of the position under LCP illumination was smaller than that under RCP illumination. The respective average dispersions under RCP and LCP illumination, σ_R_ and σ_L_, were ∼0.65 and ∼0.54 μm, respectively. This difference in the dispersion with respect to the handedness of the CPd light was attributed to the factor of Im[***H*** · E^*^] in the second term of Eq. 2. The trapping behavior of the L-form particle with respect to the polarization of the incident light had the opposite trend as shown in Fig. 2 (D and E) (σ_R_ ∼ 0.96 μm and σ_L_ ∼ 1.17 μm). The dispersion values varied with the degree of circular polarization of the incident light, defined as (*E*_L_ − *E*_R_)/(*E*_L_ + *E*_R_) (*E*_L_ and *E*_R_ are the absolute amplitudes of the LCP and RCP components of the electric field, respectively), as shown in Fig. 2 (C and F) for the D- and L-form particles, respectively. These plots of the dispersion show the opposite dependencies on the handedness of the circular polarization for the D- and L-form particles. The reversed dependencies of the D- and L-form particles can be explained by the opposite signs of Re[χ] in Eq. 2.

### Wavelength dependence of Brownian motion

**Fig. 3.**
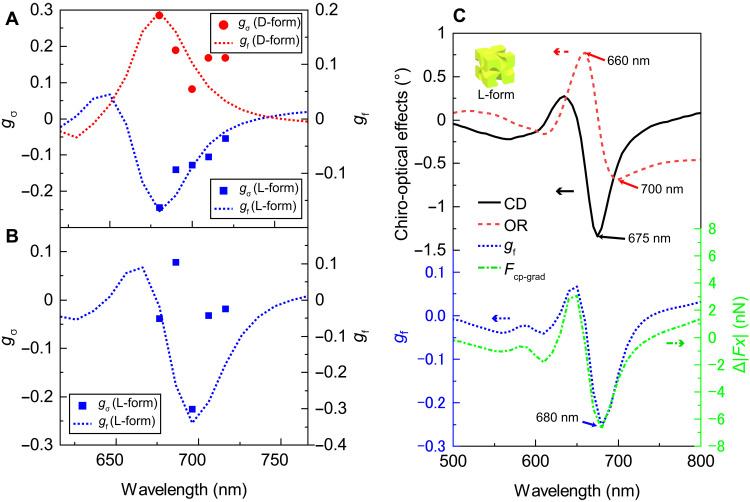
Relationship between the spectral characteristics of the gradient force and the chiro-optical effect. (**A**) Spectral features of the measured position dispersion dissymmetry (*g*_σ_; circles and squares) and the numerically simulated dissymmetry factors of the gradient force [*g*_f_ (see the text); dotted curves] for the D- and L-form chiral particles. The intensities of the incident laser were 15 and 34 mW for the D- and L-form particles, respectively. (**B**) Measured *g*_σ_ (squares) and numerically simulated *g*_f_ (dotted curves) for a large L-form nanoparticle. The intensity of the laser was 8 mW. (**C**) Simulated spectral feature of *g*_f_ and the force difference between LCP and RCP illumination (Δ∣*Fx*∣), as well as a comparison with the simulated CD and OR spectra.

The observed CP-dependent gradient force was expected to be correlated with the chiro-optical characteristics of the trapped particle (*13*). To clarify the relationship between the chiro-optical effect and the gradient force, we observed the wavelength dependence of the Brownian motion of the particles between 680 and 720 nm and plotted the difference in the position dispersion between LCP and RCP illumination. The result is shown in Fig. 3A. The dispersion differences between LCP and RCP illumination (σ_L_ − σ_R_) were normalized by −(σ_L_ + σ_R_)/2, which defines the dissymmetry factor of the position dispersion, *g*_σ_ = −2(σ_L_ − σ_R_)/(σ_L_ + σ_R_), at each wavelength. For both the D- and L-form particles, the largest difference was found at 680 nm (the shortest wavelength that our laser can provide), which approximately corresponds to the CD spectral peaks of the particles. The absolute values of *g*_σ_ decrease as the wavelength gets longer. To further clarify the relationship between the spectral characteristics of the gradient force and the chiro-optical effect, we synthesized slightly larger L-form particles (base length of ∼200 nm), with the CD peak at a longer wavelength (see the Supplementary Materials), and investigated the wavelength-dependent trapping characteristics. The *g*_σ_ value is plotted as a function of the wavelength in Fig. 3B. The *g*_σ_ value has a negative peak (*g*_σ_ ∼ −0.3) at ∼700 nm, which is close to the negative CD peak position (see the Supplementary Materials for the CD spectrum).

To analyze the behavior shown in Fig. 3 (A and B), we numerically simulated the dissymmetry factor for the gradient force, defined as the difference between the forces under LCP and RCP illumination normalized by their average, *g*_f_ = 2(∣*Fx*_L_∣ − ∣*Fx*_R_∣)/(∣*Fx*_L_∣+∣*Fx*_R_∣). The simulation was based on the finite element method (see the Supplementary Material for the details of the simulation process). Note that *g*_f_ approximately corresponds to *g*_σ_ when it is assumed that the particle is trapped in a harmonic potential. The simulated wavelength dependence curves of *g*_f_ reproduce the experimental *g*_σ_ curves of both the L- and D-form particles well, as shown in Fig. 3A, and qualitatively reproduces the *g*_σ_ curve for the larger L-form particle (Fig. 3B) when the simulated *g*_f_ curve is slightly shifted to the longer wavelength side. Then, we simulated the CD and OR with the same model and compared the results with *g*_f_, as shown in Fig. 3C. The difference in the gradient force between LCP and RCP illumination (Δ∣*Fx*∣ = ∣*Fx*_L_∣ − ∣*Fx*_R_∣) is also plotted in the figure. Here, the CD was evaluated in the simulation as the difference between the absorption of the particle (corresponding to the imaginary part of the complex refractive index) under LCP illumination and that under RCP illumination. Similarly, the OR was evaluated on the basis of the real part of the complex refractive index of the particle. Note that the spectral features of the simulated *g*_f_ (blue dashed curve) and Δ∣*Fx*∣ (green dot-dashed curve) follow the simulated CD spectrum (black solid curve) rather than the OR spectrum (red broken curve). Considering that OR and CD are correlated with the real part and imaginary part of the refractive index, respectively, and that the nonchiral gradient force is proportional to the real part of the refractive index, this finding for the simulated *g*_f_ (and Δ∣*Fx*∣) is counterintuitive. The same trend was observed with another simulation algorithm, the discrete dipole approximation method (see fig. S8).

## DISCUSSION

As this observation is relevant to an essential factor for the optical forces on chiral particles, we discuss the possible physical origins of this observation based on the analytical formulae of the gradient force. On the basis of Eq. 2, the force difference between LCP and RCP illumination can be represented by Eq. 3

$$\Delta |\mathrm{Fx}|=|Fx_{L}|-|Fx_{R}|=\Delta |\frac{1}{2}\text{Re}[\chi ]\nabla \text{Im}[H\cdot E*]|$$ (3)

In the preceding studies, only the real part of the cross-term polarizability (Re[χ]) in Eq. 3 was considered (*13*). However, we should also consider the Im [***H*** · ***E*** ^*^] in Eq. 3. ***H*** and ***E*** in this factor should be rather the total field at the position of the dipole (particle) than the incident field.

In macroscopic CD and OR spectroscopy, the signals are considered to be proportional to the imaginary and real parts of the complex refractive index, which are correlated with Im[χ] and Re[χ], respectively (see the Supplementary Materials). Thus, the observation that the wavelength dependence of Δ∣*Fx*∣ resembles the CD spectrum is most likely due to the wavelength dependence of the factor ∇Im[***H*** · E^*^]. The electromagnetic field at the position of the particle has spatial characteristics distinct from the incident field due to the existence of a chiral particle resonant with the incident field. Hence, the modification of the factor ∇Im[***H*** · E^*^] due to the particle is expected to affect the wavelength dependence of the chiral optical force. This effect is thought to be the primary reason for the observation that the chiral gradient force exhibited spectral characteristics similar to CD rather than OR. This point should be noted when discussing optical forces and optical manipulation of chiral materials, as it is a basic characteristic. More detailed discussion on this point based on the electromagnetic simulation is given in section S4.1.3 and fig. S9.

In summary, we successfully observed the CP-dependent optical gradient force exerted on chiral gold nanoparticles. The CP-dependent gradient force dominated at the wavelength where the particle had the most chiro-optical activity. In particular, for the investigated chiral gold nanoparticles, a CP-dependent force was observed either in the wavelength region where a large CD signal was observed or in a slightly redshifted region. This result differs from that of conventional CP-independent gradient forces, where the force is determined by the real part of the polarizability, which is closer to the (real part of the) refractive index than the absorption of the particle. The novel findings on the CP-dependent gradient force presented here provide a basis for the discussion of optical forces on chiral materials and introduce a new optical approach for manipulating and separating chiral materials with CPd light.

## MATERIALS AND METHODS

For the measurements of the Brownian motion of the chiral gold nanoparticles, we used a home-build optical trapping system, as illustrated in Fig. 1E. The laser source for the trapping was a Chameleon Ultra II (Coherent Inc.), which provided tunable wavelength pulses with ∼100 fs in width. To avoid the nonlinear effect of optical trapping, which appears as a repulsive gradient force under the use of femtosecond lasers (*28*), we stretched the pulse width to over several hundreds of picoseconds by a grating pair. The beam was collimated to a diameter of less than 2 mm by passing through a beam expander (a combination of ×10 and ×100 objective lenses), a λ/4 wave plate, and an objective lens for the focusing as shown in Fig. 1E.

The objective lens used for focusing had a low numerical aperture (NA = 0.20) and long working distance (WD = 50 mm). The spot size was ∼3 μm. We adopted such a weak focusing condition for the following reasons: (i) When particles are trapped with a tightly focused laser beam, the trapped particles melt and stick on the glass cell. If we try to trap the particles with the low laser power to avoid the melting while keeping the tightly focusing condition, the particle easily go away from the focusing spot due to the weak gradient force, and we cannot obtain enough long measurement time to evaluate the Brownian motion characteristics. (ii) To evaluate the relatively weak CP-dependent gradient force (<***F***_cp−grad_>), the large trapping area and the long measurement time is preferable. The CP-dependent gradient force is estimated to be nearly ∼10 times smaller than the CP-independent gradient force (<***F***_cp−grad_>) for the chiral gold nanoparticles. Therefore, the weak focusing condition is essential to increase the measurement sensitivity and detect the CP-dependent force.

The trapped particle was illuminated from the side of the cell by LEDs, and the scattered light from the trapped particle was imaged on a CMOS camera (pco.1200, PCO AG) after passing thorough an objective lens (NA = 0.9, WD = 1 mm) and a short-pass filter, which is used for the elimination of the trapping laser light. The frame rate of the CMOS camera was 25 Hz, and the measurement time to evaluate the Brownian motion characteristics of the particles was ∼12 min. The colloidal water solution of the chiral gold nanoparticles were contained in a glass cell. The density of the particles was ∼10^11^ m^−3^. The dispersions of the position in *x* and *y* directions (σ*_x_*, σ*_y_*) were calculated from the histogram of the positions projected to *x* and *y* axes by the Gaussian fitting, respectively. The Gaussian function used for the fitting was as follows

$$A\;\text{exp}\;(-{|r-r_{0}|}^{2}/2\sigma^{2})$$ (4)

where *A*, σ, and *r*_0_ (*r*_0_ = *x*_0_, *y*_0_) are the fitting parameters. *r* represents the position in *x* or *y* direction in the trapping plane (*r* = *x*, *y*).

## Supplementary Materials

### This PDF file includes:

Sections S1 to S6 Figs. S1 to S13 References
