# Supplementary material for: Optical gradient force on chiral particles
Source: Sci Adv. 2022 Sep 21;8(38):eabq2604. doi: 10.1126/sciadv.abq2604 (PMC9491721; doi:10.1126/sciadv.abq2604)
Supplement: Supplementary file 1 — Sections S1 to S6 Figs. S1 to S13 References [file sciadv.abq2604_sm.pdf]

Science Advances
AAAS

Supplementary Materials for

Optical gradient force on chiral particles

Junsuke Yamanishi *et al.*

Corresponding author: Junsuke Yamanishi, yamanishi@ims.ac.jp; Hiromi Okamoto, aho@ims.ac.jp

Sci. Adv. **8**, eabq2604 (2022)
DOI: 10.1126/sciadv.abq2604

This PDF file includes:

Sections S1 to S6
Figs. S1 to S13
References

Supplementary Material

1 Scanning electron micrographs of chiral gold nanoparticles

We used scanning electron microscope (SEM) (JSM-6700F, JEOL) with 5.0 keV electron energy for the SEM observation of the particles. The images of the D- and L-form chiral gold nanoparticles are shown in Figs. S1a, b and c, d, respectively. The image of the larger L-form chiral gold nanoparticles is shown in Fig. S2. From SEM images, we evaluate the average size of the particle as 193 and 204 nm, respectively.

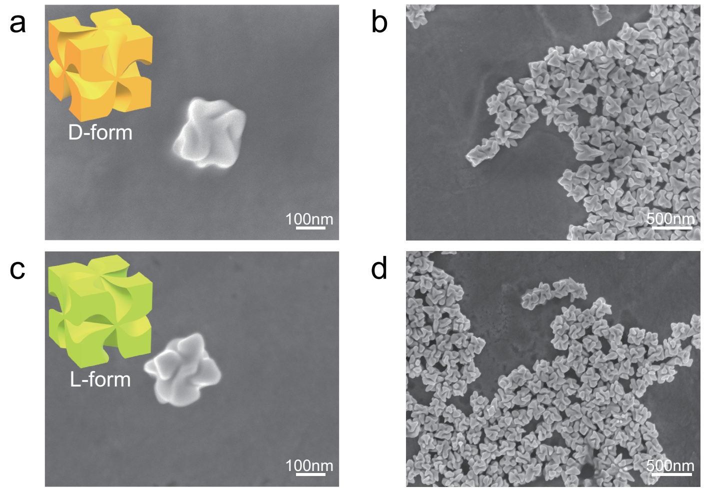

Figure S1: **SEM images of the D- and L-form chiral gold nanoparticles.** (a, b) The D-form chiral gold nanoparticles. (c, d) The L-form chiral gold nanoparticles. (a, c) show larger scale magnification images than those of (b, d).

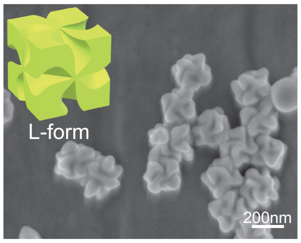

Figure S2: **SEM images of the larger L-form chiral gold nanoparticles.**

2 Spectroscopic measurement

The CD and absorption spectra of the chiral gold particle solution with the density of $\sim 2.8 \times 10^{15}$ m^{-3} were measured by commercial circular dichroism spectrometer (J-1500, JASCO Corp.) using a 1-mm path-length quartz cell. The OR spectra were evaluated by the Kramers-Krönig transformation from the measured CD spectra (*29*). The CD spectrum of the larger L-form particle discussed in Fig. 3b is shown in Fig. S3. A negative peak was found at ~ 690 nm. The width for this CD band is found to be broader than that of the smaller particles shown in Fig. 1d. The broader bandwidth is attributed to the larger non-uniformity for the larger particles in solution.

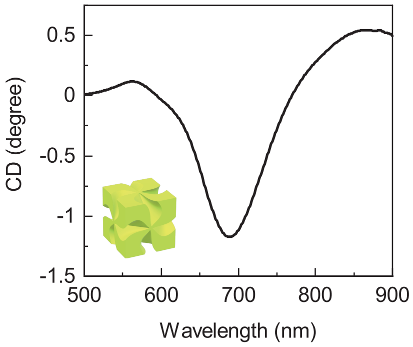

Figure S3: **The CD spectrum of the larger L-form particle discussed in Fig. 3b of the main text.**

3 The difference of the position dispersion among the individual chiral particles

The CD spectra of the particles measured by the spectrometer are the average of all particles in the solution. However, the each particle should show individual optical activity because the synthesized particles are not completely uniform (we can see the shape differences in Fig. S1b and d) (*30*). Due to the non-uniformity of the particles, CD and OR spectra exhibit broader peaks than that of individual particles. The particle-by-particle difference thus appear in the trapping force, and each particle shows different values of g_σ. As an example, Fig. S4 represents the Brownian motion of an L-form particle under the illumination of LCP and RCP light (690 nm, 48 mW). By comparing the result of Fig. S4 with that of Fig. 2 in the main text, it turns out that the difference between the Brownian motion under the LCP illumination and that under RCP illumination is quite dependent on individual particles. This is attributable to the non-uniformity of the particle shape (which reflects on χ of Eqs. (1) to (3) in the main text). Although the non-uniformity of the particles is unavoidable, the tendency shown in Figs. 2c and f in the main text were qualitatively maintained for the particles with the same handedness.

Figure S5 shows the trend of the g_σ factor for L-form particles with an incident beam at 680 nm. P_i ($i = 1, 2, 3 \cdots$) are the identifiers of particles. Although the magnitudes of g_σ factors vary for individual particles, the values are roughly $-0.1 \sim -0.2$. This difference among individual particles can be attributed mainly to the inhomogeneity of the particles. Also, g_σ factors are slightly different for measurements using the same particle. This may be due to the fact that the particles are not perfectly isotropic and the orientations of the particles relative to the incident light are not always the same between the measurements, and/or due to other unexpected measurement errors.

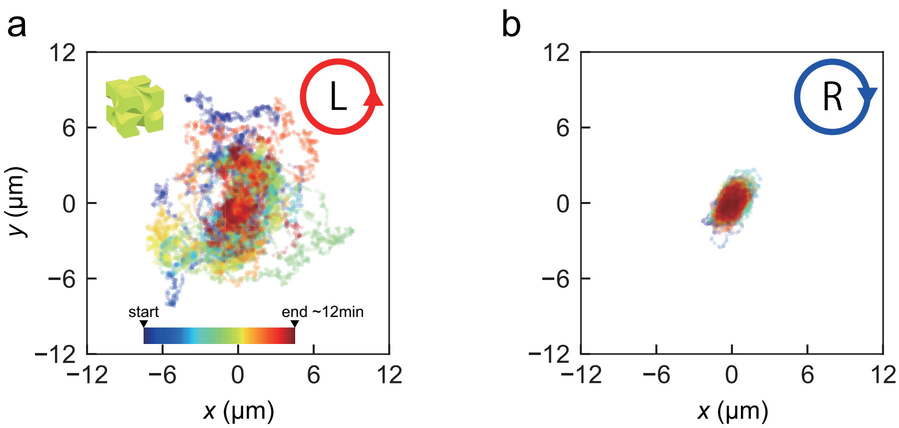

Figure S4: **The Brownian motion distribution of an L-form particle with LCP (a) and RCP (b) illumination, respectively.** The wavelength and the power of the incident laser were 690 nm and 48 mW, respectively.

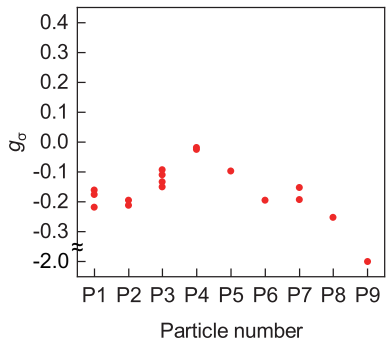

Figure S5: **Trend of the g_σ for the L-form chiral nanoparticles.** The wavelength of the incident laser for trapping was 680 nm. The lateral axis indicates the identifiers of the particles. The plural points for the same particle indicate the different measurements with the same particle.

4 Electromagnetic simulation

4.1 Simulation with finite element method

4.1.1 Procedure of simulation of the optical gradient force

We simulated the CP-dependent gradient force using a finite element method (FEM) package software COMSOL multiphysics 5.4. We first simulated the CD spectrum of the chiral nanoparticle from the calculated absorption cross-section ($C_{\rm abs}$) of the particle. It should be noted that the experimental CD spectra were obtained by measuring the extinction spectra for LCP and RCP light, which is not directly related to $C_{\rm abs}$. In the present study, we simulate the CD spectrum from $C_{\rm abs}$; CD (in degrees) $= 180 \log_{\rm e}(10) Ql(C_{\rm abs,L} - C_{\rm abs,R})/(4\pi)$, where Q $(= 2.8 \times 10^{15}\ {\rm m}^{-3})$ and l $(= 1$ mm) denote the number density of the nanoparticles and the optical path length of the cell, respectively. As is seen from the comparison between Fig. 1d and Fig. 3c in the main text, the simulated CD and OR spectra reproduced the experimental ones in a qualitatively satisfactory level.

The model of the simulation is shown in Fig. S6, where the chiral gold nanoparticle is placed at $x = x_0$. For the simulation of the CD spectrum, the chiral particle with the base length of

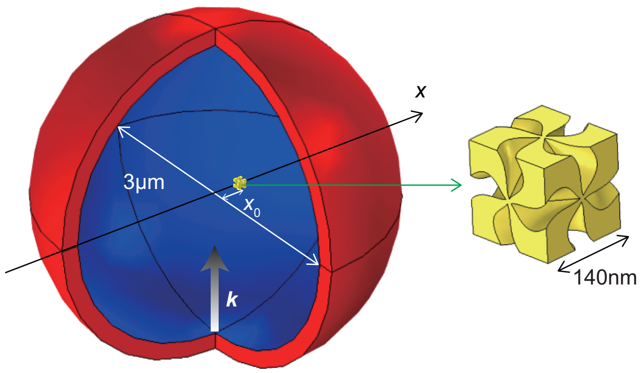

Figure S6: **The model for the simulation.** The red part is the perfect matched layer, and the blue part is the medium (water).

140 nm was placed at the center ($x_0 = 0$ nm) in water. This base length was set a little smaller than the observed one, to reproduce the spectral peaks closer to the experimental ones. The incident light was a plane wave with a power of 10^{10} Wm^{-2}. The CD peak position depended on the size and the shape of the edges of the particle. We obtained the OR spectrum from the calculated CD spectrum using the Kramers-Krönig transformation (*29*). The simulated CD and OR spectra shown in Fig. 3 of the main text was evaluated in this procedure.

Next, we discuss the gradient force with the electromagnetic simulation by the Maxwell's stress tensor. To directly evaluate the gradient force in the simulation, we assumed that the incident beam was unfocused plane wave with a gaussian distribution of intensity, ($I(r) = I_0/\sqrt{2\pi\rho^2}\mathrm{exp}(-r^2/(2\rho^2)), r^2 = x^2 + y^2$), and the particle was placed at $x_0 = 500$ nm (as in Fig. S6), and estimated the force exerted on the particle. The intensity at the center of the beam I_0 and the beam radius ρ was set to be $I_0 = 10^{10}$ Wm^{-2} and $\rho = 3.0$ μm, respectively. This model approximately reproduces the experimental condition of the trapping with the loosely focused incident beam.

We discussed in the main text the g_σ factor of the position dispersion difference for the Brownian motion ($g_\sigma = 2(\sigma_\mathrm{L} - \sigma_\mathrm{R})/(\sigma_\mathrm{L} + \sigma_\mathrm{R})$). If the potential originating from the gradient force is isotropic in the x-y plane, i.e., the focal spot is an isotropic gaussian shape, the CP-dependent gradient force, which exert on the particle near the focusing spot center, is expressed as $\boldsymbol{F} = \boldsymbol{r}/\varsigma_\mathrm{i}, (\boldsymbol{r} = [x, y], \mathrm{i} = \mathrm{L}, \mathrm{R})$ (*22*). Here, ς_i is the inverse of the spring constant of the force that is correlated with the position dispersion of the Brownian motion σ_i as $\sigma_\mathrm{i}^2 = k_\mathrm{B}T/\varsigma_\mathrm{i}$. Under this condition, $g_\mathrm{f} = -2(|Fx_\mathrm{L}|-|Fx_\mathrm{R}|)/(|Fx_\mathrm{L}|+|Fx_\mathrm{R}|) \sim g_\sigma = 2(\sigma_\mathrm{L}-\sigma_\mathrm{R})/(\sigma_\mathrm{L}+\sigma_\mathrm{R})$ is expected, which was confirmed experimentally in Figs. 3a and b in the main text.

4.1.2 CP-dependent optical gradient force: dependence on the position and the orientation of the particle

We checked through the simulation whether the approximation $g_{\mathrm{f}} \sim g_{\sigma}$ is valid, and whether the calculated force is the gradient force with the potential minimum at the spot center. Figure S7 shows the plots of the calculated trapping force Fx at the CD peak wavelength (680 nm) as functions of x_0, where the red squares and the blue circles represent those for the LCP and RCP illuminations, respectively. In both polarization conditions, the trapping forces increase Fx nearly linearly with x_0. This feature indicates that the calculated Fx can be regarded as the gradient force. The gradient force for the RCP illumination is stronger than that for the LCP illumination. This result is consistent with the experimental result (Fig. 2 in the main text). The linear dependence guarantee that the gradient force exerted on the particle is in the region of the approximation $g_{\mathrm{f}} \propto g_{\sigma}$ discussed above, under the present experimental condition. As is seen in Fig. 2 of the main text, the particle positions under the Brownian motion were in the most part found to be in the area within 500 nm from the center.

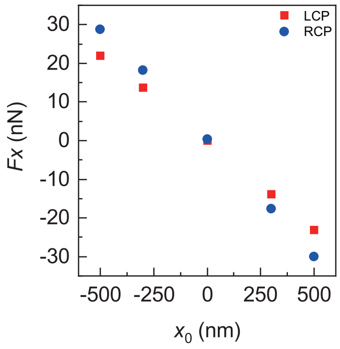

Figure S7: **Position dependence of the gradient force ($\boldsymbol{Fx}$) exerted on the chiral nanoparticle.** Red squares and blue circles represent the forces for the LCP and the RCP illuminations, respectively.

We also evaluated g_{f} of the particle in various orientation conditions as shown in Fig. S8, where ϕ and θ denote azimuth and polar angles, respectively. In all orientation, g_{f} have the same

trend, i.e. negative peak at 680 nm. This means that the CP-dependent gradient force shows essentially the same spectral feature for any orientation of the trapped particle.

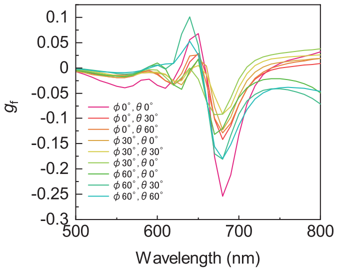

Figure S8: **Orientation dependence of $g_{\mathbf{f}}$.** ϕ and θ denote azimuth and polar angles.

4.1.3 On the wavelength dependence of calculated CP-dependent gradient force: consideration with a point dipole model

To get insight into the CP-dependent optical gradient force, we consider a point dipole model combined with the result of the FEM method. The CP-dependent optical gradient force is expressed as $< \boldsymbol{F}_{\text{cp}-\text{grad}} >= -\text{Re}[\chi]\nabla\text{Im}[\boldsymbol{H} \cdot \boldsymbol{E}^*]/2$ where the particle is assumed as a point dipole (Eq. (2) in the main text). In our model, the factor of $\text{Im}[\boldsymbol{H} \cdot \boldsymbol{E}^*]$ at the position of the point dipole (the center of the nanoparticle) is evaluated by the integral of $\text{Im}[\boldsymbol{H} \cdot \boldsymbol{E}^*]$ over the particle volume, divided by the volume of the particle. Figure S9a show the spectra of CD, OR, and $\Delta\text{Im}[\boldsymbol{H} \cdot \boldsymbol{E}^*]$ for the particle, where $\Delta\text{Im}[\boldsymbol{H} \cdot \boldsymbol{E}^*]$ represents the difference between the integrated $\text{Im}[\boldsymbol{H} \cdot \boldsymbol{E}^*]$ for LCP and that for RCP. The positive peak wavelength of $\Delta\text{Im}[\boldsymbol{H} \cdot \boldsymbol{E}^*]$ nearly matched with the main negative peak of CD. Figure S9b shows the plots of $\text{OR} \cdot \Delta\text{Im}[\boldsymbol{H} \cdot \boldsymbol{E}^*]$ and $\Delta|Fx|$. As the OR spectrum follows $\text{Re}[\chi]$, we calculated $\text{OR} \cdot \Delta\text{Im}[\boldsymbol{H} \cdot \boldsymbol{E}^*]$ as a factor that is correlated with the CP-dependent optical gradient force. The spectrum of $\text{OR} \cdot \Delta\text{Im}[\boldsymbol{H} \cdot \boldsymbol{E}^*]$ follows well that of $\Delta|Fx|$, and shows positive and negative peaks at the close wavelengths. Although the FEM calculation is not rigorously consistent with the model of point dipole because the particle has finite volume in the FEM calculation, this

discussion indicates that the spectral feature of chiral optical force is related to not only $\mathrm{Re}[\chi]$ but also the factor of $\mathrm{Im}[\boldsymbol{H} \cdot \boldsymbol{E}^*]$.

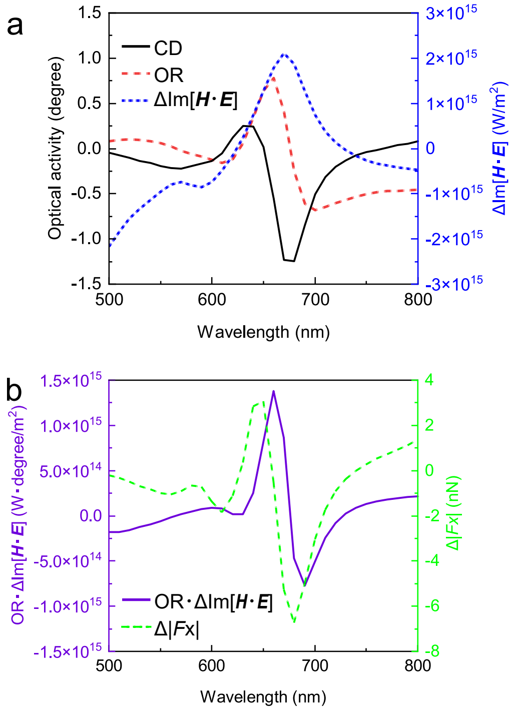

Figure S9: **Simulated CP-dependent optical gradient force in a model described in the text.** (a) Plots of CD, OR, and $\Delta\mathrm{Im}[\boldsymbol{H} \cdot \boldsymbol{E}^*]$ against the wavelength for the chiral gold nanoparticle. (b) Plots of $\mathrm{OR} \cdot \Delta\mathrm{Im}[\boldsymbol{H} \cdot \boldsymbol{E}^*]$, and $\Delta|Fx|$ against the wavelength.

4.1.4 Effects of other terms of the optical force

Based on Ref. (*10*), there are 8 terms that can influence the optical force exerting on the trapped particle as shown in Eq. (S1). Among those terms, we discuss the first term as the origin of the chiral effects of the trapping force.

$$< \boldsymbol{F} >= \nabla U + \sigma \frac{< \boldsymbol{S} >}{c} - \mathrm{Im}[\chi]\nabla \times < \boldsymbol{S} > + c\sigma_{\mathrm{e}} \nabla \times < \boldsymbol{L}_{\mathrm{e}} > + c\sigma_{\mathrm{m}} \nabla \times < \boldsymbol{L}_{\mathrm{m}} >$$
$$+ \omega\gamma_e < \boldsymbol{L}_{\mathrm{e}} > + \omega\gamma_{\mathrm{m}} < \boldsymbol{L}_{\mathrm{m}} > + \frac{ck_0^4}{12\pi}\mathrm{Im}[\alpha\beta^*]\mathrm{Im}[\boldsymbol{E} \times \boldsymbol{H}^*], \quad \text{(S1)}$$

where $U = (\mathrm{Re}[\alpha]|\boldsymbol{E}|^2 + \mathrm{Re}[\beta]|\boldsymbol{H}|^2 - 2\mathrm{Re}[\chi]\mathrm{Im}[\boldsymbol{H}\cdot\boldsymbol{E}^*])/4$ is the term due to particle–field interaction; $<\boldsymbol{S}>= \mathrm{Re}[\boldsymbol{E}\times\boldsymbol{H}^*]/2$ is the time-averaged Poynting vector; $<\boldsymbol{L}_{\mathrm{e}}>= \frac{\epsilon_0}{4\omega i}\boldsymbol{E}\times\boldsymbol{E}^*$ and $<\boldsymbol{L}_{\mathrm{m}}>= \frac{\mu_0}{4\omega i}\boldsymbol{H}\times\boldsymbol{H}^*$ are the time-averaged electric and magnetic spin densities, respectively; $\sigma_{\mathrm{e}} = k_0\mathrm{Im}[\alpha]/\epsilon_0$, and $\sigma_{\mathrm{m}} = k_0\mathrm{Im}[\beta]/\mu_0$ are the electric and magnetic absorption cross-sections ($\sigma = \sigma_{\mathrm{e}} + \sigma_{\mathrm{m}}$), respectively; $\gamma_{\mathrm{e}} = -2\omega\mathrm{Im}[\chi] + ck_0^4\mathrm{Re}[\alpha\chi^*]/(3\pi\epsilon_0)$ and $\gamma_{\mathrm{m}} = -2\omega\mathrm{Im}[\chi] + ck_0^4\mathrm{Re}[\beta\chi^*]/(3\pi\epsilon_0)$ also have the dimension of a cross-section. α, β, and χ denote the electric, magnetic, and cross-term (electric-magnetic) polarizabilities of the particle, respectively. We discuss here the effects of the other seven terms on optical force. The magnetic spin density ($<\boldsymbol{L}_{\mathbf{m}}>$) is small compared with the contribution of its electric counterpart $<\boldsymbol{L}_{\mathbf{e}}>$ because non-magnetic materials give a relatively stronger response to the electric field, and $\beta = 0$ for non-magnetic materials. Therefore, the fifth, seventh, and last terms in Eq. (S1) are negligible. Further, the electric spin density appear in the condition that the particle is on the high reflective substrate such as gold or high refractive index material (*10*). In our measurement condition, the interface between the substrate (glass) and water has quite small reflection at the interface between water and glass. We simulated $<\boldsymbol{L}_{\mathbf{e}}>$ assuming the particle center is at $x = 500$ nm as in Fig. S6, and the result is shown in Fig. S10a and b with LCP and RCP incident light, respectively. The wavelength of the incident light was 680 nm. Color density and black arrows in the figures represent x component of the electric spin density ($<\boldsymbol{L}_{\mathbf{e}}>_x$) and $y - z$ components of that ($[<\boldsymbol{L}>_y, <\boldsymbol{L}>_z]$), respectively. $<\boldsymbol{L}_{\mathbf{e}}>_x$ show both positive and negative values in the particle almost symmetrically. The symmetrical distribution result in the zero value of total $<\boldsymbol{L}_{\mathbf{e}}>_x$ in the particle and the sixth term in Eq. (S1) can be negligible. Moreover, the black arrows show approximately symmetrical distribution, which lead to the small rotation of that around the particle, and the fourth term also vanish as discussed in Ref. (*10*). Next, we consider the possibility of second and third terms. We show $<\boldsymbol{S}>$ in Fig. S10c, and d at $x = 500$ nm with LCP and RCP incident light, respectively. Color den-

sity and black arrows in the figure represent x component of the time-averaged Poynting voctor ($< \boldsymbol{S} >_x$) and $y - z$ components of that ($[< \boldsymbol{S} >_y, < \boldsymbol{S} >_z]$), respectively. Considering the distribution of these figures, as the same reason as the fourth and sixth terms of Eq. (S1), the second and third terms can be negligible. Finally, we map the distribution of $\mathrm{Im}[\boldsymbol{H} \cdot \boldsymbol{E}^*]$ in Fig. S10e and f with with LCP and RCP incident light, respectively. The distribution show almost uniformly negative values in the interior part of the particle. Therefore, the non-negligible $\mathrm{Im}[\boldsymbol{H} \cdot \boldsymbol{E}^*]$ generates the chiro-optical gradient force, which is included in the first term of Eq. (S1).

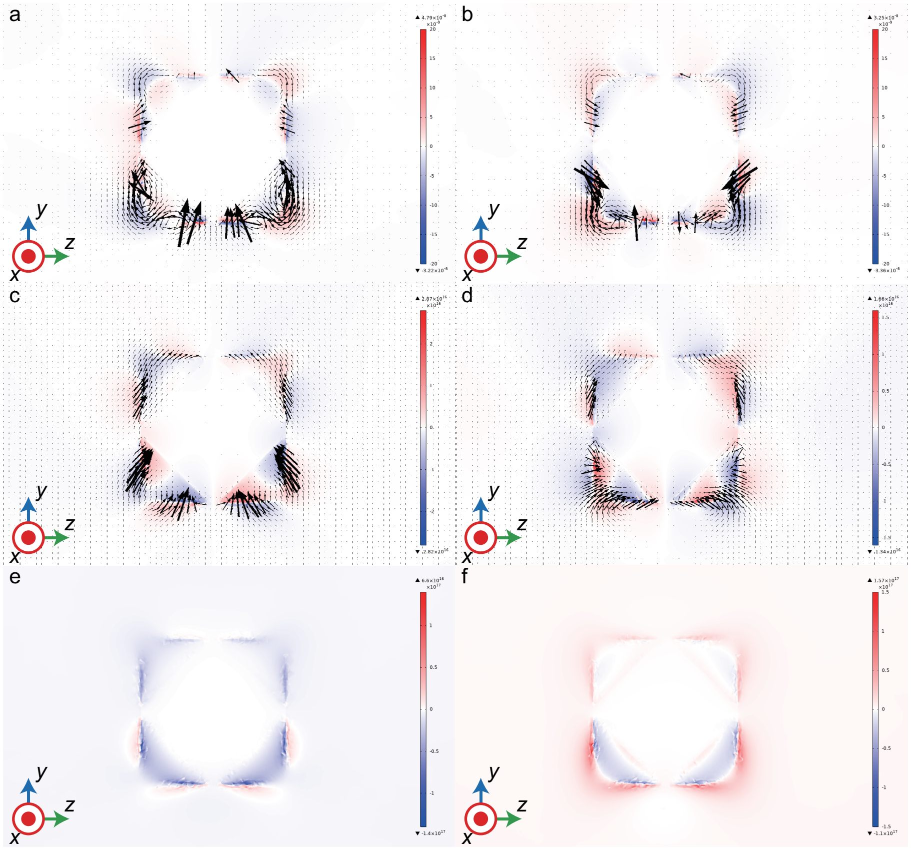

Figure S10: **Mapping of electric spin density, Poynting vector, and optical chirality of the field.** (a, b) the time-averaged electric spin density ($< \boldsymbol{L}_\mathbf{e} >$), (c, d) the time-averaged Poynting vector ($< \boldsymbol{S} >$), and (e, f) optical chirality ($\mathrm{Im}[\boldsymbol{H} \cdot \boldsymbol{E}^*]$). (a, c, e) are the maps with LCP illumination, and (b, d, f) are those with RCP illumination. Color density and black arrows in the figures represent x components and $y - z$ components (vector). The wavelength of the incident light was 680 nm.

4.2 Simulation with discrete dipole approximation method

To confirm the result of FEM simulation, we repeated the simulation of the optical spectra and the optical force under the same condition with a different numerical method, discrete dipole approximation (DDA). The simulated optical spectra are plotted in Fig. S11a. The wavelength of negative peak in the CD spectrum is found at 640 nm which is slightly blue-shifted compared with the result of FEM (675 nm), due to the difference of the calculation method. However, the essential features of the CD, OR, and absorption spectra are the same as those obtained with FEM. The g_{f} spectrum obtained with DDA, shown in Fig. S11b, has a negative peak near the wavelength of the CD negative peak. The agreement with the FEM results confirms the finding that the peak of g_{f} for this chiral particles appears around the peak of CD spectrum.

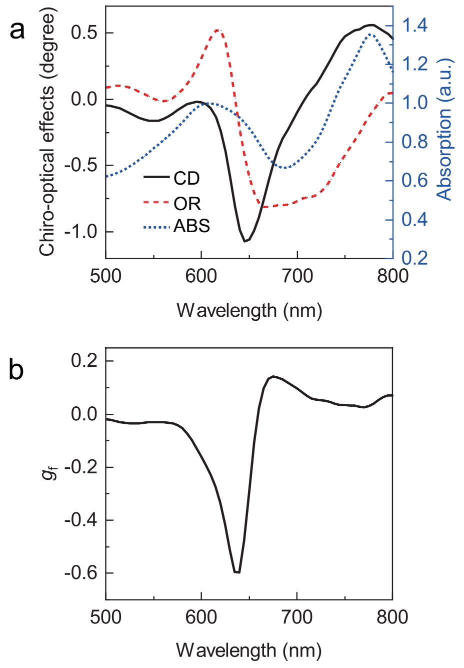

Figure S11: **Simulated result of CD, OR, and absorption (a) spectra, and g_{f} spectrum (b).**

4.3 FEM and DDA simulation for a simple two orthogonal gold bars model structure

We further checked the reproducibility of the simulation results with the two different algorishm on a simple model structure consists of two orthogonal gold bars (see Fig. S12). The optical behavior of this model system has been sometimes treated with the analytical models in the past. The rectangular geometry of the model is favorable for the calculation with DDA. The simulated results of CD, OR, and force difference ($\Delta Fx = Fx_{\mathrm{L}} - Fx_{\mathrm{R}}$) in both methods are shown in Figs. S13a, b. The absorption spectra were calculated as $(C_{\mathrm{abs,L}} + C_{\mathrm{abs,R}})/2$ $(10^{-10} \times \mathrm{cm}^2)$. In all cases for CD, OR, and ΔFx, the both methods gave the same trends to each other. Although the spectra of ΔFx in this model structure rather resemble the OR spectra than the CD spectra, ΔFx spectra have the split peaks at 670 and 710 nm in both simulation methods. The inconsistent features between the simulated ΔFx and OR indicate that the chiral gradient force is not directly correlated with the real part of the polarizability of the chiral particles even in this simple model.

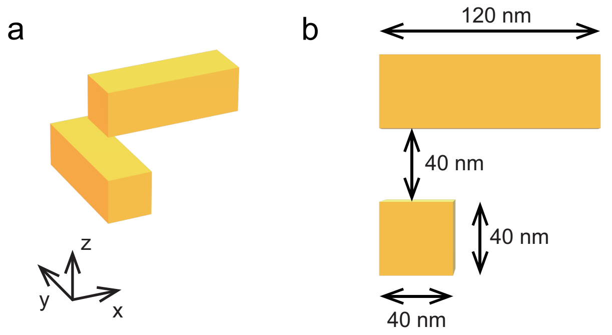

Figure S12: **Two orthogonal gold bars model.** (a) over view. (b) side view toward $+y$.

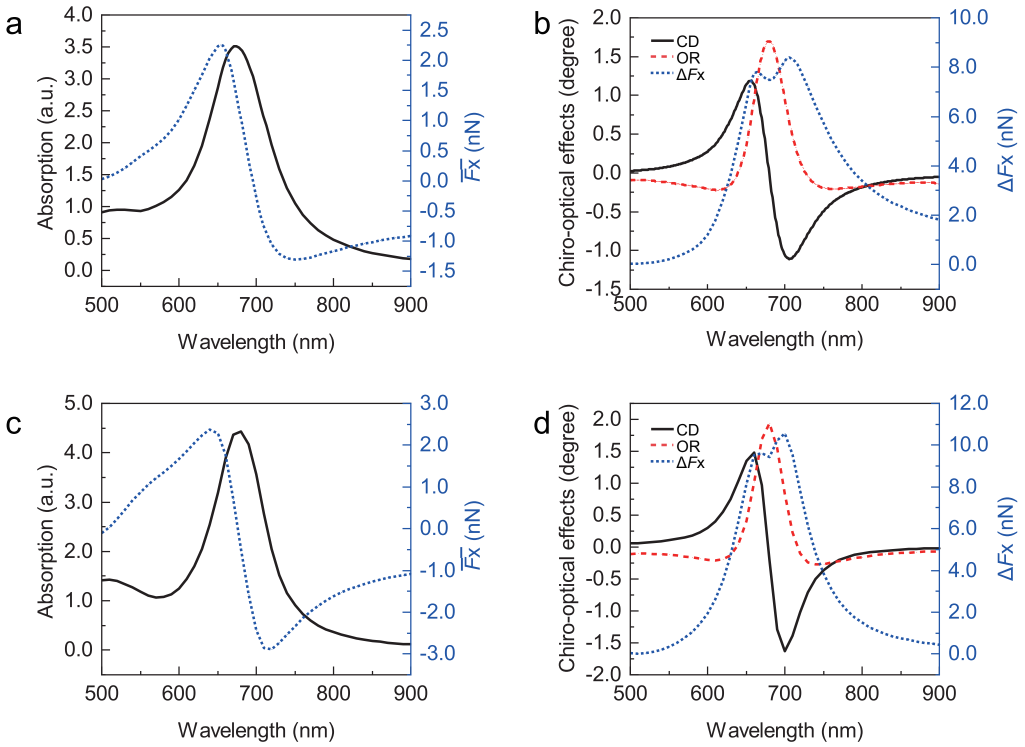

Figure S13: **Comparison of the results of electromagnetic simulation with the different methods (FEM (a, b) and DDA (c, d)).** (a, c) Spectra of absorption and averaged force in LCP and RCP ($\overline{Fx} = (Fx_{\mathrm{L}} + Fx_{\mathrm{R}})/2$). (b, d) Spectra of CD, OR and force difference (ΔFx).

5 Theoretical considerations of the relation between the optical force and the optical activity

Here, we analytically describe that the CP-dependent force is influenced by chiro-optical features of chiral materials.

5.1 Chiro-optial effects

The chiral constitutive equations for the chiral materials is given as follows (*31, 32*).

$$\boldsymbol{D} = \epsilon_0\epsilon_\mathrm{r}\boldsymbol{E} + i\xi\boldsymbol{B} \tag{S2}$$

$$\boldsymbol{H} = \frac{1}{\mu_0\mu_\mathrm{r}}\boldsymbol{B} + i\eta\boldsymbol{E}, \tag{S3}$$

where ϵ_r and μ_r are the relative permittivity and permeability of the material, respectively, ξ and η denote the chiral parameters, and ϵ_0 and μ_0 are the permittivity and the permeability of vacuum, respectively. We assume, in this paper, that the anisotropy of the particle is small, and the permittivity, the permeability, and the chiral parameters are treated as scalar quantities for simplicity. From the Maxwell's equation, we obtain the following equations if the applied field has a single frequency ω.

$$\nabla \times \boldsymbol{E} = -\frac{\partial \boldsymbol{B}}{\partial t} = i\omega\boldsymbol{B}, \tag{S4}$$

$$\nabla \times \boldsymbol{H} = \frac{\partial \boldsymbol{D}}{\partial t} = -i\omega\boldsymbol{D}. \tag{S5}$$

Considering Eq. (S4), Eq. (S2) is transformed into

$$\boldsymbol{D} = \epsilon_0\epsilon_\mathrm{r}\boldsymbol{E} + i\xi\boldsymbol{B} = \epsilon_0\epsilon_\mathrm{r}\boldsymbol{E} + \frac{\xi}{\omega}\nabla \times \boldsymbol{E}. \tag{S6}$$

From Eq. (S3), (S5), and (S6), $\nabla \times \boldsymbol{B}$ is transformed as

$$\begin{aligned}\nabla \times \boldsymbol{B} &= \mu_0\mu_\mathrm{r}\nabla \times (\boldsymbol{H} - i\eta\boldsymbol{E}) = \mu_0\mu_\mathrm{r}(-i\omega\boldsymbol{D} - i\eta\nabla \times \boldsymbol{E}) \\ &= \mu_0\mu_\mathrm{r}\{-i\omega(\epsilon_0\epsilon_\mathrm{r}\boldsymbol{E} + \frac{\xi}{\omega}\nabla \times \boldsymbol{E}) - i\eta\nabla \times \boldsymbol{E}\} = -i\mu_0\mu_\mathrm{r}\{\omega\epsilon_0\epsilon_\mathrm{r}\boldsymbol{E} + (\xi + \eta)\nabla \times \boldsymbol{E}\}.\end{aligned} \tag{S7}$$

The electric field of the light propagating toward the z-direction is given as

$$\boldsymbol{E} = \boldsymbol{E_0} \exp\left(-i\omega t + i\boldsymbol{k}\cdot\boldsymbol{r}\right) = \begin{bmatrix} E_x \\ E_y \\ E_z \end{bmatrix} \exp\left(-i\omega t + ikz\right), \tag{S8}$$

where $\boldsymbol{k} = [0, 0, k]$ is the wavenumber. Then, we have

$$\nabla \times \boldsymbol{E} = \begin{bmatrix} -E_y \\ E_x \\ 0 \end{bmatrix} ik \exp\left(-i\omega t + ikz\right) = \begin{bmatrix} 0 & -ik & 0 \\ ik & 0 & 0 \\ 0 & 0 & 0 \end{bmatrix} \boldsymbol{E}. \tag{S9}$$

From Eq. (S4),

$$\begin{aligned}
\nabla \times \nabla \times \boldsymbol{E} &= -\frac{\partial \nabla \times \boldsymbol{B}}{\partial t} = -\frac{\partial}{\partial t}\left[-i\mu_0\mu_\mathrm{r}\{\omega\epsilon_0\epsilon_\mathrm{r}\boldsymbol{E} + (\xi+\eta)\nabla\times\boldsymbol{E}\}\right] \\
&= \omega\mu_0\mu_\mathrm{r}\{\omega\epsilon_0\epsilon_\mathrm{r}\boldsymbol{E} + (\xi+\eta)\nabla\times\boldsymbol{E}\} \\
&= \omega\mu_0\mu_\mathrm{r}\left\{\omega\epsilon_0\epsilon_\mathrm{r}\begin{bmatrix} 1 & 0 & 0 \\ 0 & 1 & 0 \\ 0 & 0 & 1 \end{bmatrix} + (\xi+\eta)\begin{bmatrix} 0 & -ik & 0 \\ ik & 0 & 0 \\ 0 & 0 & 0 \end{bmatrix}\right\}\boldsymbol{E} \\
&= \frac{\omega^2}{c^2}\left\{\epsilon_\mathrm{r}\mu_\mathrm{r}\begin{bmatrix} 1 & 0 & 0 \\ 0 & 1 & 0 \\ 0 & 0 & 1 \end{bmatrix} + \begin{bmatrix} 0 & -ik\mu_0\mu_\mathrm{r}(\xi+\eta)\frac{c^2}{\omega} & 0 \\ ik\mu_0\mu_\mathrm{r}(\xi+\eta)\frac{c^2}{\omega} & 0 & 0 \\ 0 & 0 & 0 \end{bmatrix}\right\}\boldsymbol{E} \\
&= \frac{\omega^2}{c^2}\left\{\epsilon_\mathrm{r}\mu_\mathrm{r}\begin{bmatrix} 1 & 0 & 0 \\ 0 & 1 & 0 \\ 0 & 0 & 1 \end{bmatrix} + \begin{bmatrix} 0 & -iNc\mu_0\mu_\mathrm{r}(\xi+\eta) & 0 \\ iNc\mu_0\mu_\mathrm{r}(\xi+\eta) & 0 & 0 \\ 0 & 0 & 0 \end{bmatrix}\right\}\boldsymbol{E} \\
&= \frac{\omega^2}{c^2}\begin{bmatrix} \zeta_{xx} & \zeta_{xy} & 0 \\ -\zeta_{xy} & \zeta_{xx} & 0 \\ 0 & 0 & \zeta_{xx} \end{bmatrix}\boldsymbol{E} = \frac{\omega^2}{c^2}\tilde{\zeta}\boldsymbol{E}.
\end{aligned} \tag{S10}$$

Here, we used Eq (S7), (S8), and (S9) for the transformation of the equations, and we used $c^2 = 1/\epsilon_0\mu_0$ and $N = ck/\omega$, where c is the velocity of light in a vacuum.

In the Generality, $\nabla \times \boldsymbol{A}\exp\left(-i\omega t + i\boldsymbol{k}\cdot\boldsymbol{r}\right) = i\boldsymbol{k}\times\boldsymbol{A}\exp\left(-i\omega t + i\boldsymbol{k}\cdot\boldsymbol{r}\right)$. Hence, (S10) is converted into

$$\boldsymbol{k}\times\boldsymbol{k}\times\boldsymbol{E} = (\boldsymbol{E}\cdot\boldsymbol{k})\boldsymbol{k} - |\boldsymbol{k}|^2\boldsymbol{E} = -\frac{\omega^2}{c^2}\tilde{\zeta}\boldsymbol{E}. \tag{S11}$$

If we introduce the complex refractive index vector, $\boldsymbol{N} = N\boldsymbol{k}/|\boldsymbol{k}|$, Eq. (S11) becomes

$$N^2\boldsymbol{E} - (\boldsymbol{E}\cdot\boldsymbol{N})\boldsymbol{N} - \tilde{\zeta}\boldsymbol{E} = 0. \tag{S12}$$

The second term in Eq. (S12) is 0 because $\boldsymbol{N} \perp \boldsymbol{E}$ for the propagating light. The eigen equation (S12) is then expressed as follows,

$$\begin{bmatrix} N^2-\zeta_{xx} & -\zeta_{xy} & 0 \\ \zeta_{xy} & N^2-\zeta_{xx} & 0 \\ 0 & 0 & N^2-\zeta_{xx} \end{bmatrix} \begin{bmatrix} E_x \\ E_y \\ E_z \end{bmatrix} = 0. \tag{S13}$$

To solve this equation, we utilize the fact that N must fulfill the following equation,

$$(N^2-\zeta_{xx})^2 + \zeta_{xy}^2 = 0. \tag{S14}$$

Here, $\zeta_{xx} = \epsilon_{\mathrm{r}}\mu_{\mathrm{r}}$ and $\zeta_{xy} = -iNc\mu_0\mu_r(\xi+\eta)$ from Eq. (S10). Now, the solution to Eq. (S16) is given as

$$N^2 = \zeta_{xx} \pm i\zeta_{xy} = \epsilon_{\mathrm{r}}\mu_{\mathrm{r}} \pm c\mu_0\mu_r(\xi+\eta)N = a \pm bN. \tag{S15}$$

Here, we separate the solution of N for an above equation as $N_\pm = n_\pm + i\kappa_\pm$. N_+ and N_- correspond to the complex refractive indices for LCP and RCP light, respectively. $N_\pm$ is divided into real and imaginary parts as $N_\pm = n_\pm + i\kappa_\pm$, and

$$N_\pm^2 = (n_\pm + i\kappa_\pm)^2 = n_\pm^2 - \kappa_\pm^2 + 2in_\pm\kappa_\pm, \tag{S16}$$

We note that each of N_+ and N_- has two solutions. However, the solution with $\kappa_\pm < 0$ is physically incorrect because the light intensity increases with the propagation in the material. Now

$$\begin{aligned} a \pm bN_\pm &= \mathrm{Re}[a] + i\mathrm{Im}[a] \pm (\mathrm{Re}[b] + i\mathrm{Im}[b])(n_\pm + i\kappa_\pm) \\ &= \{\mathrm{Re}[a] \pm (\mathrm{Re}[b]n_\pm - \mathrm{Im}[b]\kappa_\pm)\} + i\{\mathrm{Im}[a] \pm (\mathrm{Re}[b]\kappa_\pm + \mathrm{Im}[b]n_\pm)\}. \end{aligned} \tag{S17}$$

Then,

$$n_\pm^2 - \kappa_\pm^2 = \mathrm{Re}[a] \pm (\mathrm{Re}[b]n_\pm - \mathrm{Im}[b]\kappa_\pm), \tag{S18}$$

$$2in_\pm\kappa_\pm = i\{\mathrm{Im}[a] \pm (\mathrm{Re}[b]\kappa_\pm + \mathrm{Im}[b]n_\pm)\}. \tag{S19}$$

Considering Eq. (S18),

$$(n_+^2 - \kappa_+^2) - (n_-^2 - \kappa_-^2) = (\mathrm{Re}[b]n_+ - \mathrm{Im}[b]\kappa_+) + (\mathrm{Re}[b]n_- - \mathrm{Im}[b]\kappa_-) \tag{S20}$$

Here, we define

$$n = (n_+ + n_-)/2, \kappa = (\kappa_+ + \kappa_-)/2, \tag{S21}$$

$$\Delta n = (n_+ - n_-), \Delta\kappa = (\kappa_+ - \kappa_-). \tag{S22}$$

Then, from Eq. (S20),

$$n\Delta n - \kappa\Delta\kappa = \mathrm{Re}[b]n - \mathrm{Im}[b]\kappa, \tag{S23}$$

and from Eq. (S19),

$$2n_+\kappa_+ - 2n_-\kappa_- = \mathrm{Re}[b](\kappa_+ + \kappa_-) + \mathrm{Im}[b](n_+ + n_-), \tag{S24}$$

and with Eq. (S21) and (S22)

$$n\Delta\kappa + \kappa\Delta n = \kappa\mathrm{Re}[b] + n\mathrm{Im}[b]. \tag{S25}$$

From Eq. (S23), (S25) and (S16), we have

$$\Delta n = \mathrm{Re}[b] = \mathrm{Re}[c\mu_0\mu_\mathrm{r}(\xi + \eta)], \tag{S26}$$

$$\Delta\kappa = \mathrm{Im}[b] = \mathrm{Im}[c\mu_0\mu_\mathrm{r}(\xi + \eta)]. \tag{S27}$$

The CD and the OR spectra correspond to $\Delta\kappa$ and Δn, respectively.

5.2 Optical force

We consider the macroscopic polarization $\boldsymbol{P}$ of chiral materials expressed as follows to estimate the optical gradient force, on the assumption that $\eta = \xi$, as sometimes adopted for isotropic chiral materials (*33, 34*), and $\mu_\mathrm{r} = 1$.

$$\boldsymbol{P} = \boldsymbol{D} - \epsilon_0\boldsymbol{E} = \epsilon_0(\epsilon_\mathrm{r} - 1)\boldsymbol{E} + \frac{\xi}{\omega}\nabla\times\boldsymbol{E} = \epsilon_0\begin{bmatrix} \epsilon_r - 1 & -ik\mu_0\xi\frac{c^2}{\omega} & 0 \\ ik\mu_0\xi\frac{c^2}{\omega} & \epsilon_r - 1 & 0 \\ 0 & 0 & \epsilon_r - 1 \end{bmatrix}\boldsymbol{E}$$
$$= \epsilon_0\begin{bmatrix} \zeta_{xx} - 1 & \zeta_{xy}/2 & 0 \\ -\zeta_{xy}/2 & \zeta_{xx} - 1 & 0 \\ 0 & 0 & \zeta_{xx} - 1 \end{bmatrix}\boldsymbol{E}. \quad \text{(S28)}$$

Here, we considered that the light is propagating in the z-direction.

Now we consider an isolated particle. The polarization of an isolated particle can be then represented as

$$\boldsymbol{p} = \boldsymbol{P}/Q = \frac{\epsilon_0}{Q}\begin{bmatrix} \zeta_{xx} - 1 & \zeta_{xy}/2 & 0 \\ -\zeta_{xy}/2 & \zeta_{xx} - 1 & 0 \\ 0 & 0 & \zeta_{xx} - 1 \end{bmatrix}\boldsymbol{E} = \frac{\epsilon_0}{Q}(\zeta_{xx} - 1)\boldsymbol{E} - \frac{\epsilon_0\zeta_{xy}}{2ikQ}\nabla\times\boldsymbol{E}$$
$$= \alpha\boldsymbol{E} + i\chi\boldsymbol{H}, \quad \text{(S29)}$$

where Q is the number density of particles that generate the macroscopic polarization $\boldsymbol{P}$, and α and χ the electric and the cross-term polarizability.

Note that $\epsilon_0(\zeta_{xx} - 1)/Q$ and $-\zeta_{xy}/(2NcQ)$ approximately equal to α and $i\chi$, respectively. From this relation and Eqs. (S26) and (S27), we can understand that Δn and $\Delta\kappa$ are proportional to the real and imaginary parts of χ, respectively ($\mathrm{Re}[\chi] \propto \mathrm{Re}[\xi + \eta] \propto \Delta n$). When we neglect the effect of the magnetic polarizability, the gradient force exerted on a single chiral particle is represented as (*10, 12*),

$$< \boldsymbol{F}_\mathrm{grad} > = \frac{1}{4}\nabla(\mathrm{Re}[\alpha]|\boldsymbol{E}|^2 - 2\mathrm{Re}[\chi]\mathrm{Im}[\boldsymbol{H}\cdot\boldsymbol{E}^*]). \quad \text{(S30)}$$

Hence, the CP-dependent gradient force is represented as follows, by the use of Eq. (S29),

$$< \boldsymbol{F}_{\mathrm{cp-grad}} >= -\frac{1}{2}\mathrm{Re}[\chi]\nabla\mathrm{Im}[\boldsymbol{H}\cdot\boldsymbol{E}^*] \sim -\frac{\mu_0\mathrm{Re}[\xi]}{2Q}\nabla\mathrm{Im}[\boldsymbol{H}\cdot\boldsymbol{E}^*] \propto \Delta n. \tag{S31}$$

This means that the CP-dependent gradient force is correlated with optical rotation Δn under the assumption mentioned above.

6 Heat effect

The energy of the incident light is partially absorbed by the particle and converted to heat, which possibly have some effects on the mechanical behavior of the particle. We estimate here how the temperature of the particle rises under the present experimental condition. Previous research on the temperature of the gold nanoparticle with radius of 75 nm near the glass substrate suggested that the temperature rise was about 300 K under $10\ \mathrm{mW}\mu\mathrm{m}^{-2}$ illumination at the wavelength where the particle show $3.2 \times 10^{-15}\ \mathrm{m}^2$ for the absorption cross-section (*35*). In our gold particle, the effective radius was ~ 118 nm and the power density was only $\sim 0.53\ \mathrm{mW}\mu\mathrm{m}^{-2}$, and the difference of the absorption cross-section between LCP and RCP at the wavelength of 680 nm was $1.4 \times 10^{-15}\ \mathrm{m}^2$. The temperature rise is thus estimated to be only ~ 2.9 K at the center of the focal spot (*36*). That is only less than $1\ \%$ of the thermal energy at room temperature that determines the Brownian motion. This effect is at least an order of magnitude smaller than the chiro-optical effect of the trapping, and such an effect is negligible in our experimental condition.

REFERENCES AND NOTES

1. A. Rodger, B. Nordén, *Circular Dichroism and Linear Dichroism*, vol. 1 (Oxford Univ. Press, 1997).

2. N. Berova, K. Nakanishi, R. W. Woody, *Circular Dichroism: Principles and Applications* (John Wiley & Sons, 2000).

3. L. D. Barron, *Molecular Light Scattering and Optical Activity* (Cambridge Univ. Press, 2009).

4. M. Schäferling, Chiral nanophotonics: Chiral optical properties of plasmonic systems, in *Springer Series in Optical Sciences* (Springer International, 2017), vol. 205, pp. 159.

5. E. Hendry, T. Carpy, J. Johnston, M. Popland, R. V. Mikhaylovskiy, A. J. Lapthorn, S. M. Kelly, L. D. Barron, N. Gadegaard, M. Kadodwala, Ultrasensitive detection and characterization of biomolecules using superchiral fields, *Nat. Nanotechnol.* **5**, 783–787 (2010).

6. A. Ben-Moshe, S. G. Wolf, M. B. Sadan, L. Houben, Z. Fan, A. O. Govorov, G. Markovich, Enantioselective control of lattice and shape chirality in inorganic nanostructures using chiral biomolecules, *Nat. Commun.* **5**, 4302 (2014).

7. Z. Fan, A. O. Govorov, Chiral nanocrystals: Plasmonic spectra and circular dichroism, *Nano Lett.* **12**, 3283–3289 (2012).

8. N. J. Greenfield, Using circular dichroism spectra to estimate protein secondary structure, *Nat. Protoc.* **1**, 2876–2890 (2006).

9. R. Hassey, E. J. Swain, N. I. Hammer, D. Venkataraman, M. D. Barnes, Probing the chiroptical response of a single molecule, *Science* **314**, 1437–1439 (2006).

10. S. B. Wang, C. T. Chan, Lateral optical force on chiral particles near a surface, *Nat. Commun.* **5**, 3307 (2014).

11. Y. Zhao, A. A. E. Saleh, J. A. Dionne, Enantioselective optical trapping of chiral nanoparticles with plasmonic tweezers, *ACS Photonics* **3**, 304–309 (2016).

12. Y. Zhao, A. A. E. Saleh, M. A. van de Haar, B. Baum, J. A. Briggs, A. Lay, O. A. Reyes-Becerra, J. A. Dionne , Nanoscopic control and quantification of enantioselective optical forces, *Nat. Nanotechnol.* **12**, 1055 (2017), 1059.

13. A. Canaguier-Durand, J. A. Hutchison, C. Genet, T. W. Ebbesen, Mechanical separation of chiral dipoles by chiral light, *New J. Phys.* **15**, 123037 (2013).

14. G. Tkachenko, E. Brasselet, Spin controlled optical radiation pressure, *Phys. Rev. Lett.* **111**, 033605 (2013).

15. G. Tkachenko, E. Brasselet, Optofluidic sorting of material chirality by chiral light, *Nat. Commun.* **5**, 3577 (2014).

16. S. Albaladejo, M. I. Marqués, M. Laroche, J. J. Sáenz, Scattering forces from the curl of the spin angular momentum of a light field, *Phys. Rev. Lett.* **102**, 113602 (2009).

17. R. P. Cameron, S. M. Barnett, A. M. Yao, Discriminatory optical force for chiral molecules, *New J. Phys.* **16**, 013020 (2014).

18. H. Chen, Y. Jiang, N. Wang, W. Lu, S. Liu, Z. Lin, Lateral optical force on paired chiral nanoparticles in linearly polarized plane waves, *Opt. Lett.* **40**, 5530–5533 (2015).

19. M. Li, S. Yan, Y. Zhang, Y. Liang, P. Zhang, B. Yao, Optical sorting of small chiral particles by tightly focused vector beams, *Phys. Rev. A* **99**, 033825 (2019).

20. H. Zheng, H. Chen, J. Ng, Z. Lin, Optical gradient force in the absence of light intensity gradient, *Phys. Rev. B* **103**, 035103 (2021).

21. H.-E. Lee, H.Y. Ahn, J. Mun, Y. Y. Lee, M. Kim, N. H. Cho, K. Chang, W. S. Kim, J. Rho, K. T. Nam, Amino-acid-and peptide-directed synthesis of chiral plasmonic gold nanoparticles, *Nature* **556**, 360–365 (2018).

22. P. M. Hansen, V. K. Bhatia, N. Harrit, L. Oddershede, Expanding the optical trapping range of gold nanoparticles, *Nano Lett.* **5**, 1937–1942 (2005).

23. L. Novotny, B. Hecht, *Principles of Nano-Optics* (Cambridge Univ. Press, 2012).

24. K. Svoboda, S. M. Block, Optical trapping of metallic rayleigh particles, *Opt. Lett.* **19**, 930–932 (1994).

25. M. L. Juan, M. Righini, R. Quidant, Plasmon nano-optical tweezers, *Nat. Photonics* **5**, 349–356 (2011).

26. N. H. Cho, G. H. Byun, Y.C. Lim, S. W. Im, H. Kim, H.E. Lee, H.Y. Ahn, K. T. Nam, Uniform chiral gap synthesis for high dissymmetry factor in single plasmonic gold nanoparticle, *ACS Nano* **14**, 3595–3602 (2020).

27. Z. Yan, J. Sweet, J. E. Jureller, M. J. Guffey, M. Pelton, N. F. Scherer, Controlling the position and orientation of single silver nanowires on a surface using structured optical fields, *ACS Nano* **6**, 8144–8155 (2012).

28. Y. Jiang, T. Narushima, H. Okamoto, Nonlinear optical effects in trapping nanoparticles with femtosecond pulses, *Nat. Phys.* **6**, 1005–1009 (2010).

29. L. A. Nafie, *Vibrational Optical Activity: Principles and Applications* (John Wiley & Sons, 2011).

30. J. Karst, N. H. Cho, H. Kim, H.E. Lee, K. T. Nam, H. Giessen, M. Hentschel, Chiral scatterometry on chemically synthesized single plasmonic nanoparticles, *ACS Nano* **13**, 8659–8668 (2019).

31. K. Cho, *Reconstruction of Macroscopic Maxwell Equations*, vol. 237 (Springer, 2018).

32. K. Sakoda, *Electromagnetic Metamaterials: Modern Insights Into Macroscopic Electromagnetic Fields*, vol. 287 (Springer Nature, 2019).

33. A. Sihvola, Electromagnetic modeling of BI-isotropic media, *Prog. Electromagn. Res.* **9**, 45–86 (1994).

34. T. G. Mackay, A. Lakhtakia, *Electromagnetic Anisotropy and Bianisotropy: A Field Guide* (World Scientific, 2009).

35. K. Setoura, S. Ito, H. Miyasaka, Stationary bubble formation and marangoni convection induced by CW laser heating of a single gold nanoparticle, *Nanoscale* **9**, 719–730 (2017).

36. Y. Seol, A. E. Carpenter, T. T. Perkins, Gold nanoparticles: Enhanced optical trapping and sensitivity coupled with significant heating, *Opt. Lett.* **31**, 2429–2431 (2006).
